# Comparing interventions and exploring neural mechanisms of exercise in Parkinson disease: a study protocol for a randomized controlled trial

**DOI:** 10.1186/s12883-015-0261-0

**Published:** 2015-02-05

**Authors:** Gammon M Earhart, Ryan P Duncan, John L Huang, Joel S Perlmutter, Kristen A Pickett

**Affiliations:** Program in Physical Therapy, Washington University School of Medicine, St. Louis, MO USA; Department of Neurology, Washington University School of Medicine, St. Louis, MO USA; Department of Anatomy and Neurobiology, Washington University School of Medicine, St. Louis, MO USA; Department of Radiology, Washington University School of Medicine, St. Louis, MO USA; Occupational Therapy Program, University of Wisconsin, Madison, WI USA

**Keywords:** Parkinson disease, Gait, Exercise, Magnetic resonance imaging, Functional connectivity, Rehabilitation

## Abstract

**Background:**

Effective treatment of locomotor dysfunction in Parkinson disease (PD) is essential, as gait difficulty is an early and major contributor to disability. Exercise is recommended as an adjunct to traditional treatments for improving gait, balance, and quality of life. Among the exercise approaches known to improve walking, tango and treadmill training have recently emerged as two promising therapies for improving gait, disease severity and quality of life, yet these two interventions have not been directly compared to each other. Prior studies have been helpful in identifying interventions effective in improving gait function, but have done little to elucidate the neural mechanisms underlying functional improvements. The primary objective of the proposed work is to compare the effects of three community-based exercise programs, tango, treadmill training and stretching, on locomotor function in individuals with PD. In addition, we aim to determine whether and how these interventions alter functional connectivity of locomotor control networks in the brain.

**Methods/Design:**

One hundred and twenty right-handed individuals with idiopathic PD who are at least 30 years of age will be assigned in successive waves to one of three community-based exercise groups: tango dancing, treadmill training or stretching (control). Each group will receive three months of exercise training with twice weekly one-hour group classes. Each participant will be evaluated at three time points: pre-intervention (baseline), post-intervention (3 months), and follow-up (6 months). All evaluations will include assessment of gait, balance, disease severity, and quality of life. Baseline and post-intervention evaluations will also include task-based functional magnetic resonance imaging (fMRI) and resting state functional connectivity MRI. All MRI and behavioral measures will be conducted with participants OFF anti-Parkinson medication, with behavioral measures also assessed ON medication.

**Discussion:**

This study will provide important insights regarding the effects of different modes of exercise on locomotor function in PD. The protocol is innovative because it: 1) uses group exercise approaches for all conditions including treadmill training, 2) directly compares tango to treadmill training and stretching, 3) tests participants OFF medication, and 4) utilizes two distinct neuroimaging approaches to explore mechanisms of the effects of exercise on the brain.

**Trial registration:**

ClinicalTrials.gov NCT01768832.

## Background

Parkinson disease (PD) is the second most common neurodegenerative disorder in the United States, affecting 1–1.5 million Americans [1]. Numerous non-motor and motor symptoms including gait dysfunction characterize PD. Gait dysfunction frequently causes substantial and early difficulties in people with PD and may represent the leading edge of disability [2]. Reduced speed and stride length contribute to gait dysfunction [3,4]. These gait difficulties not only affect forward walking, but also impair backward walking, during which gait speed and stride length are reduced to a greater degree relative to healthy controls [5,6]. While pharmacological and surgical approaches to the management of PD can help to partially alleviate some gait problems, they do not completely address the issue, indicating a need for additional and complementary approaches to the treatment of gait in PD. Numerous studies demonstrated the effectiveness of exercise as a complementary treatment for improving gait function in PD. Among the exercise approaches known to improve walking function, tango and treadmill training have recently emerged as two promising therapies for improving gait while also reducing disease severity and improving quality of life (for reviews see [7-9]), yet tango and treadmill training have not been directly compared to each other. Furthermore, the neural mechanisms underlying these interventions remains to be elucidated. Neuroimaging may be a means to investigate these underlying mechanisms

Investigations of gait-related dysfunction in humans is particularly challenging. Neuroimaging may provide a means to investigate the role of exercise interventions in individuals with PD, but this approach presents a challenge due to difficulties collecting neuroimaging data during a gait task. Alternatively, imaging during an imagined walking task can provide insights into underlying brain mechanisms related to gait in healthy controls [10,11] and those with neurological disorders [12,13]. This imagined task activates networks involved with actual walking [10]. Another neuroimaging method that can be used to investigate brain networks related to locomotion networks uses resting state functional connectivity magnetic resonance imaging (rs-fcMRI), which assesses brain activity at rest and therefore eliminates the need to evoke actual or imagined locomotor behaviors. In contrast to task-related activations, rs-fcMRI focuses on spontaneous fluctuations in the blood oxygen level dependent (BOLD) signal at rest; regional fluctuations in these signals correlate with functionally related regions. Thus, rs-fcMRI identifies related networks within the resting state BOLD data, revealing functionally related resting state networks that can be quantified. [14]. These rs-fcMRI methods identify common networks in healthy individuals and can detectchanges in these networks related to a variety of neurological disorders\(for review see [15]). Resting state-functional connectivity MRI holds great promise as a potential biomarker for progression of disease, elucidating compensatory mechanisms associated with disease processes, informing targeting of neurosurgical treatments, and investigating new treatment strategies. rs-fcMRI has not yet been applied to study the effects of exercise on functional brain networks in humans with PD.

To investigate and compare the mechanisms underlying various PD exercise training approaches, we plan to conduct a 5-year, prospective, randomized, blinded study of a cohort of persons with PD. The main objective of this study will be to determine the effects of tango, treadmill training, and stretching (control) exercise on locomotor function and other aspects of PD. Additionally, we aim to determine how exercise impacts activity of specific brain regions and networks involved in locomotion using task-based and rs-fcMRI.

We hypothesize that tango and treadmill training will show similar improvement with forward walking performance, but only tango will improve backward walking performance. Tango and treadmill training will similarly improve disease severity. Additionally, tango will result in large improvements in balance and quality of life compared to treadmill training and stretching. With neuroimaging, we hypothesize that a differential BOLD signal increase will be observed in regions of interest during imagined forward walking following tango and treadmill training, and during imagined backward walking following tango only. Additionally, following tango and treadmill training, rs-fcMRI coherence in the motor network will be increased and will correlate with improvements in gait performance. No significant rs-fcMRI or task-related BOLD signal changes will occur in the stretching group.

## Methods

### Study design

**Figure 1:**
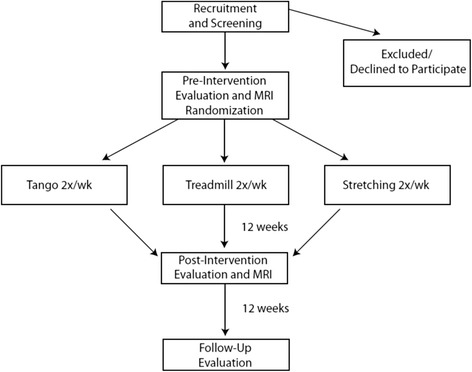
**Diagram showing the flow of participants through the study.**

This project is a 5-year, prospective, randomized controlled trial (Figure 1). Participant will be assigned to one of three different exercise groups (tango, treadmill, stretching). Assessments will be conducted at three time points: pre-intervention (baseline), post-intervention (3 months), and follow-up (6 months). The primary variable of interest is gait velocity in both forward and backward walking. Secondary variables include motor sign severity (Movement Disorders Society-Unified Parkinson Disease Rating Scale-subsection III), balance (mini-Balance Evaluation Systems Test (mini-BESTest)), and quality of life (Parkinson Disease Questionnaire-39). All evaluations will be administered by a research physical therapist. The MDS-UPDRS-III and mini-BESTest measures will be video recorded and evaluated separately by a Movement Disorders neurologist, who will be blinded to the group assignment, assessment time point, and medication status of each participant. Information on current medications and dosages will be collected at all evaluations so that changes in medication over the course of the study can be noted and controlled for during statistical analyses. Participants will also undergo separate task-based and rs-fcMRI to explore the effects of these exercise interventions on brain activity. We will focus on locomotor network activity within specific regions of interest including the premotor area, supplementary motor area, primary somatosensory cortex, putamen, and cerebellum. We will also correlate changes in connectivity with changes in motor performance. This study is approved by the Human Research Protection Office at the Washington University School of Medicine and meets all guidelines associated with human subject research. All participants will provide written and informed consent prior to participation in this study.

### Participant selection

One hundred and twenty right-handed participants with idiopathic PD who are at least 30 years of age will be recruited through the Washington University School of Medicine’s Movement Disorders Center, Volunteers for Health database, and the Greater St. Louis Area Chapter of American Parkinson Disease Association. Participants must be diagnosed with clinically defined “definite PD”, as previously described by Racette et al. [16] based upon established criteria [17-19]. Each participant must: 1) have clear benefit from levodopa, 2) be classified between Hoehn & Yahr stages I-IV, and 3) be able to walk independently with or without an assistive device for at least three meters. Participants will be randomly recruited from our community and assigned to interventions in blocks of 10–16 participants per wave. Three or four waves of each exercise group will be conducted as needed to achieve the desired sample size.

### Procedures

All physical evaluations and MRI scans will be conducted OFF medication, i.e. after a minimum 12-hour withdrawal of all anti-Parkinson medications. Evaluations for a single individual will always take place at the same time of day for all visits. MRI scans will also take place at the same time of day but on a separate date as close as possible to the associated date of evaluation. During the physical evaluation, participants will be evaluated first during the OFF medication period, given their normal dosage of anti-Parkinson medication, and then re-evaluated during the ON medication phase after a 45–60 minute waiting period to allow the medication to take effect. OFF medication measures will provide more accurate information about disease severity and effects of the disease on physical function without the confound of medication masking some symptoms, while ON medication measures will provide information about participant function in their everyday medicated state*.* During evaluations, participants will be allowed to rest as often as needed. Measures to be collected at each physical evaluation are included in Table 1. At the pre- and post-intervention time points, each participant will undergo task-based fMRI and rs-fcMRI scans as detailed below in *MRI Protocol*. Demographic information will be collected at baseline only and will include age, gender, height, weight, and duration of PD symptoms.

**Table 1:** **Outcome measures administered at each evaluation**

**Outcome measure**	**Reliability/Validity references**
Spatiotemporal Features of Gait	Nelson et al. 2002 [20]
MDS-UPDRS-3	Goetz et al. 2008 [21]
Mini-BESTest	Leddy et al. 2011 [22]
PDQ-39	Jenkinson et al. 1997 [23]

### MRI protocol

MRI data will be collected on a Siemens 3.0 T TrioTim scanner located in Mallinckrodt Institute of Radiology Neuroimaging Laboratory at the Washington University School of Medicine.

**Figure 2:**
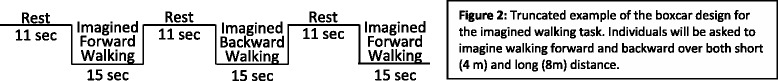
**Illustration of boxcar design for imagined walking tasks.**

During each visit participants will have 2 structural scans: a T1-weighted (T1W) sagittal, magnetization prepared rapid acquisition with gradient echo (MP-RAGE, Repetition Time (TR) =2400 ms, TI = 1000 ms, Echo Time (TE) = 3.16 ms, Flip Angle (FA) = 8°, 0.9 mm^3^ voxels, 8:09 min) and a T2-weighted (T2W) fast spin echo (TR = 3200 ms, TE = 455 ms, 1.0 mm^3^ voxels, 4:43 min). At each session, gradient and shim coil currents will be adjusted to correct for spatial inhomogeneities using the scanner’s multi-axis-projection shim program. A midsagittal scout T1W spin-echo pulse sequence will be used for positioning. Resting state-functional connectivity MRI data will be acquired via two T2*-weighted gradient echo multislice BOLD sensitized fMRI scans (Echo Planar Imaging (EPI); TR = 2200 ms, TE =3 ms, 4.0 mm^3^ voxels, 7:30-per scan, 200 frames). BOLD scans will capture 36 slices covering the brain and the cerebellum. During the rs-fcMRI scan, the participant is instructed to remain alert but relaxed with their eyes closed. Participants are monitored via an MRI-compatible eye tracker during data collection to ensure that their eyes remain closed throughout each session. Resting state data will be acquired prior to any imagined walking scans. The imagined walking component will consist of an additional two T2*-weighted gradient echo multislice BOLD sensitized fMRI scans (EPI; TR = 2200 ms, TE = 3 ms, 4.0 mm^3^ voxels, 7:30-per scan, 200 frames), acquired while the participant views a cue or null condition on a mirror mounted to the head coil. Cues will be generated via a custom made ePrime program using the design shown in Figure 2.

To ensure adherence and maximize vividness of the imagined tasks, we will utilize a variable distance cued gait task. This method has been described previously [24]. In brief, prior to beginning the scanning sessions, each individual will practice performing and imagining three tasks: walking forward, walking backward, and quiet standing. Forward and backward walking tasks will be completed at four and eight meters. Following each practiced gait task, the participant will be seated and given a stopwatch. They will then be asked to close their eyes and visualize themselves standing on the start-line. When they are able to clearly see them self in position they will press the stopwatch button and beginning imagining themselves walking the indicated distance from the first person perspective. With their eyes closed they will continue to imagine themselves walking down the hall until they cross the finish-line for the specified distance. At this point they should press the stopwatch button a second time to stop the timer.

Once in the MRI scanner, participants will be instructed to imagine walking forward or backward for four or eight meters. In addition to the walking tasks, participants will also imagine themselves standing quietly or performing a “rest”. As described previously [24,25], imagined stand trials will be collected during a separate acquisition. An fMRI compatible response pad (Mag Design and Engineering, Redwood City, CA) will be used to record the task initiation and termination times, thus allowing for assessment of task completion intervals. Any participant who does not appropriately scale their response to the change in task interval will be excluded from further analyses. Furthermore, each participant will be directly observed during each scan to check for any spurious movements of the limbs or body. If seen, then this BOLD run will be stopped and the person reminded to try to hold still.

Processing of fMRI BOLD data for the imagined walking conditions will be completed in Brain Voyager QX (Brain Innovation, Maastricht, The Netherlands) and using custom Matlab scripts (Mathworks, Inc., Natick, MA). To facilitate comparison of anatomical locations across all participants, transformation of T1 weighted scans into Talairach space will be completed prior to functional data alignment [26]. Blood oxygenated level dependent data will undergo 3D motion correction and alignment to the T1W anatomical data prior to post hoc comparisons. Comparisons between stimuli will be done using an eight predictor general linear model (GLM). The hemodynamic response function will be accounted for within the GLM model.

Processing of BOLD rs-fcMRI data will follow the methods of Fox et al. [27] and include compensation for systematic, slice-dependent time shifts, elimination of systematic odd-even slice intensity differences due to interleaved acquisition, rigid-body correction for head motion within and across runs, and normalization of the signal intensity across each run (skipping the first two frames) to obtain a whole-brain mode value of 1000. Atlas transformation will be achieved by composition of affine transforms connecting the first functional volume (averaged over all fMRI runs after cross-run realignment) with the T2W and T1W structural images. Common mode image registration (to correct for head motion within and across fMRI runs and affine warping of T1W images to our atlas image) will be done using standard algorithms. Cross-modal registration (e.g., T2W to T1W images) will be performed using vector gradient measure maximization. Linear trends across runs will be removed voxel-wise, and the data will be low-passed filtered retaining frequencies below 0.1Hz. Several sources of spurious variance will be removed by regression of the following nuisance variables and their first derivatives: (1) the 6 parameters resulting from rigid body correction for head motion; (2) a signal from a ventricular region of interest (ROI); (3) and a signal from a white matter ROI. BOLD data from each rs-fcMRI run will be resampled to 2-mm cubic voxels in atlas space for final analyses. Rigorous QA measures will be applied to minimize the impact of motion artifacts and movement confound. fMRI runs in which head motion exceeds 0.6 mm rms or the voxel-wise temporal standard deviation averaged over the brain (after full preprocessing) exceeds 0.5% will be excluded. Volumes with large changes in signal intensity (DVARS > 0.5%) will be excluded. Participants with more than 15% volumes removed also will be excluded [28].

### Intervention groups

All groups will participate in community-based group exercise, attending one-hour sessions twice per week for twelve weeks. Each class will include a brief warm up and cool down with 45 minutes of the hour devoted to the specific exercise.

### Tango

Argentine tango classes will be modeled on those from previous studies, as described in Hackney & Earhart [29,30]. Participants in the Tango class will dance both leading and following roles to ensure that everyone spends time moving forward and backward. Participants will also change partners frequently to ensure they get experience dancing with many different partners. Tango partners will be individuals without PD and will include spouses and caregivers of those with PD as well as healthy volunteers. A syllabus and standardized musical selections will be utilized to ensure that all waves of the tango class are consistent.

### Treadmill

Participants in the treadmill class will walk at their self-selected comfortable over-ground walking pace in order to exercise at intensities comparable to the tango class. Furthermore, recent evidence suggests that training at preferred speed for longer durations is more effective in enhancing gait than training at higher intensity for shorter blocks of time [31,32]. Over ground walking speed will be reassessed every week and the treadmill speed will be adjusted accordingly to ensure it matches over ground walking speed. To match the social aspect of the tango, where one interacts with a partner, treadmills will be arranged in clusters so that each individual has a walking partner with whom they can interact with during training. As with tango, people will be required to change partners frequently throughout a session. Treadmill classes will use the same musical selections as the tango classes.

### Stretching

Participants in the stretching class will be instructed by a trained physical therapist on stretching and flexibility exercises taken from the Be Active and Fitness Counts programs [33,34]. As with the other groups, each individual will be assigned a partner and will switch partners regularly during each session. Stretching classes will use the same musical selections as the tango and treadmill classes.

### Primary outcome measure

#### Gait

Spatiotemporal features of gait will be measured at each evaluation using a five-meter instrumented, computerized walkway (GAITRite, CIR Systems, Inc., Havertown, PA). The primary variable of interest from the GAITRite will be forward gait velocity. Additional variables of interest include stride length, stride length variability (CV), and gait asymmetry ([35,36]). Participants will perform three trials of each of the following walking conditions: 1) forward, 2) backward, 3) dual-task, and 4) fast as possible. Results from the three trials of each walking task will be averaged. For the dual-task condition, participants will be instructed to walk at their self-selected pace while completing a phonemic naming task. For the fast as possible condition, participants will be instructed to walk as quickly and safely as possible without running.

### Secondary outcome measures

Motor sign severity will be measured at each evaluation using the MDS-UPDRS-III questionnaire. This section consists of 18 items pertaining to the cardinal motor features of PD, with each item rated on a 5 point (0–4) ordinal scale. Higher scores indicate greater PD motor sign severity.

Balance will be measured at each evaluation using the Mini-Balance Evaluation Systems Test (Mini-BEST). This test includes 14 items addressing four balance systems: anticipatory postural adjustments, reactive postural control, sensory orientation, and dynamic gait stability. Each item is rated on a 3 point (0–2) ordinal scale. Lower scores indicate greater balance impairment.

Quality of life will be measured at each evaluation using the Parkinson Disease Questionnaire (PDQ-39), a 39-item self-report questionnaire, which assesses PD-specific health related quality over the previous month. Higher scores indicate worse quality of life.

Endurance will be measured at each evaluation using the 6-Minute Walk Test (6MWT). Participants will be instructed to walk, at a self-selected speed, as far a distance as possible over the span of 6 minutes. Participants will be allowed to use assistive devices but must stay consistent from evaluation to evaluation.

### BOLD activity in imagined walking tasks

Region of Interest (ROI) beta weight values for the general linear model contrasts (Forward vs. Rest and Backward vs. Rest) will be used to compare changes in BOLD signal values during imagined gait at the pre-test and post-test time points.

### Functional connectivity

The strength of correlations between brain regions will be used to assess changes in rs-fcMRI from pre-test to post-test.

### Statistical analysis

We will employ a repeated measure (RM) ANOVA with group (tango, treadmill, and stretching) and time (pre-intervention, post-intervention, and follow-up) as factors to determine whether and how these interventions impact gait velocity. Similar analyses will also be performed for secondary variables of interest, including the MDS-UPDRS-III, Mini-BESTest, and PDQ-39*.* To analyze fMRI BOLD data, we will apply random effects (RFX) general linear model analysis using forward, backward and standing imagined tasks as individual predictors. A false discovery rate (FDR) correction of Q = 0.01 will be used to compensate for multiple comparisons. Significant clusters of voxels will be identified for pre- and post-intervention conditions. Additionally, the three groups will be compared to each other in the post-intervention condition using RFX ANCOVA. For analysis of rs-fcMRI data we will use an independent component analysis (ICA) to examine the strength of the correlation coefficients for anatomical regions identified as part of the locomotor control network, which include the premotor area, supplementary motor area, primary somatosensory cortex, putamen, and cerebellum. The strength of these correlations before and after the intervention will be compared via two-tailed RM ANOVAs with group and time as factors. Finally, we will correlate BOLD signal changes during the imagined walking tasks and changes in connectivity with changes in motor performance using Pearson or Spearman linear correlations as appropriate. The a-priori alpha level for analyses is set at 0.05.

### Power analysis

We used PASS software (NCSS, LLC, Kaysville, UT) to calculate statistical power based on preliminary data*.* Our primary variable of interest for behavioral data is gait velocity. Average forward gait velocity was 1.2 ± 0.1 m/s and average backward gait velocity was 0.6 ± 0.1 in a sample of 60 individuals with PD (H&Y = 2.4 ± 0.2) tested OFF medication in our laboratory over the past year for a different study. The current study has been powered to be able to detect differences of 1 standard deviation, or 0.1 m/s, which is equivalent to an effect size of 0.4. Changes of 0.1 m/s or more in gait velocity have been reported previously following both tango [29,30,37] and treadmill training [9,38-40]. With an effect size of 0.4, 27 participants per group will provide 89% power to detect differences between or within groups using two-tailed tests at a significance level of 0.05*.* Based upon our previous data, this sample size is also more than sufficient to adequately power our secondary variables (MDS-UPDRS-III, Mini-BESTest, and PDQ-39).

Sample sizes for imaging portions of this study were calculated based upon preliminary data from our lab and sources in the literature. To analyze fMRI BOLD data, our preliminary data from control participants before and after a single session of treadmill training indicate an effect size of 0.91 for change in beta weights (pre = −0.094 ± 0.111, post = 0.418 ± 0.116, Earhart laboratory unpublished data) using the cerebellum as an exemplar region during imagined forward walking. We recognize that responses in those with PD may be less pronounced than in controls and as such have used a conservative effect size of 0.45, half of that observed in controls, for our power calculation. Our preliminary imagined task-based fMRI BOLD data from people with PD (n = 8) indicate a beta weight of −0.075 ± 0.097, which is comparable to the value obtained from young controls at baseline. Utilizing the data from the sample with PD and an estimated effect size of 0.45, 18 subjects per groups will provide 80% power to detect differences between groups and 88% power to detect within group differences. Significant findings of differential BOLD signal changes have been localized to specific brain regions in PD populations during similar imagined walking MRI paradigms with a total of 12 PD participants per group [41], further supporting that our proposed sample size is adequate. Utilizing rs-fcMRI to determine correlations between regions in the locomotor control network, we have powered the study to enable detection of differences in correlation coefficients of 0.1 (mean correlation = 1.0, SD = 0.1, p = 0.05, effect size = 0.44, per data from Wu et al. [42] and our own preliminary data). We will have 80% power to detect between group differences and 88% power to detect within group differences with 18 subjects per group.

Based upon our previous intervention studies [30], we anticipate an attrition rate of approximately 15-20% due to dropouts or failure to complete the required minimum of 85% of classes. We also expect MRI-related exclusions due to head or body motion in roughly 30% of tested individuals*.* As such, we will recruit 40 subjects per group to account for attrition and data loss due to head or body motion and still obtain the needed final sample sizes for each aim.

## Discussion

This study is innovative in several respects with two novel aspects that will potentially lay the foundation for many future studies. First, this will be the first study to perform a direct, head-to-head comparison of tango and treadmill training. Currently, it is unclear which of the interventions provides the largest improvement in gait, or which of these interventions may be better for improving PD motor symptoms, balance, and quality of life. This intervention study will address these questions and could lead to studies that combine these two treatment approaches into an exercise program that could be more effective than either intervention on its own. Second, the use of two distinct neuroimaging techniques, fMRI BOLD and rs-fcMRI, before and after intervention, will allow us to explore neurological mechanisms underlying changes in physical performance due to exercise training. The imaging techniques proposed herein will also lay the groundwork for us to answer many other questions regarding locomotor control mechanisms in PD.

## Abbreviations

6MWT: 6 minute walk test
ANCOVA: Analysis of covariance
BOLD: Blood oxygen level dependent
EPI: Echo planar imaging
FA: Flip angle
fMRI: Functional magnetic resonance imaging
GLM: General linear model
MDS-UPDRS III: Movement disorder society version of the unified parkinson disease rating scale, motor subscale III
Mini-BEST: Mini Balance Evaluation Systems Test
MP-RAGE: Magnetization prepared rapid acquisition with gradient echo
MRI: Magnetic resonance imaging
PD: Parkinson’s disease
PDQ-39: Parkinson’s disease questionnaire
RFX ANOVA: Random effects analysis of variance
rs-fcMRI: Resting-state functional connectivity magnetic resonance imaging
ROI: Region of interest
T1W: T1-weighted
T2W: T2-weighted
TE: Echo time
TR: Repetition time
